# A Polyclinic for Atlanta

**Published:** 1886-04

**Authors:** 


### A POLYCLINIC FOR ATLANTA.

The polyclinic of Vienna is an institution that has been in successful operation for many years. It is a place where the poorer classes of that city receive gratuitous medical advice. The work is divided into the different specialties, such as surgery, medicine, diseases of women, diseases of the skin, etc., and over each department some specialist has supervision. The chief feature of the institution is the provision for special clinical work, especially for advanced students and post-graduates. Short courses of six to eight weeks are given in each of the special departments usually to a limited number of applicants, and the work is eminently practical.

The polyclinic in America is a comparatively new affair, and is carried on according to the German idea. In many of our large cities these institutions have recently been started, and they supplement the work of the medical colleges. It is with pleasure that we announce the establishment of such an institution in Atlanta, and under the management of the efficient corps of physicians who will direct its affairs, a good work will be accomplished. It is proposed to organize and systematize the medical out-door work of Atlanta. There will be six departments, presided over by Drs. A. W. Calhoun, J. S. Todd, W. S. Elkin, G. H. Noble, A. J. Woodward and Henry Wile.

The Atlanta polyclinic starts out under most favorable auspices, and it may be assured of our most hearty co-operation and support.

